# Supplementary material for: Structure-guided functional suppression of AML-associated DNMT3A hotspot mutations
Source: Nat Commun. 2024 Apr 10;15:3111. doi: 10.1038/s41467-024-47398-y (PMC11006857; doi:10.1038/s41467-024-47398-y)
Supplement: Supplementary file 1 — Supplementary information [file 41467_2024_47398_MOESM1_ESM.pdf]

Supplementary Information

Structure-guided functional suppression of AML-associated DNMT3A hotspot mutations

Jiuwei Lu[1,7], Yiran Guo[2,3,7], Jiekai Yin[4], Jianbin Chen[1], Yinsheng Wang[4,5], Gang Greg Wang[2,3,6,*], Jikui Song[1,4,*]

[1]Department of Biochemistry, University of California, Riverside, CA 92521, USA

[2]Department of Pharmacology and Cancer Biology, Duke University School of Medicine, Durham, NC 27710, USA

[3]Duke Cancer Institute, Duke University School of Medicine, Durham, NC 27710, USA

[4]Environmental Toxicology Graduate Program, University of California, Riverside, CA 92521, USA

[5]Department of Chemistry, University of California, Riverside, CA 92521, USA

[6]Department of Pathology, Duke University School of Medicine, Durham, NC 27710, USA

[7]These authors contributed equally to this work

*Correspondence: greg.wang@duke.edu; jikui.song@ucr.edu

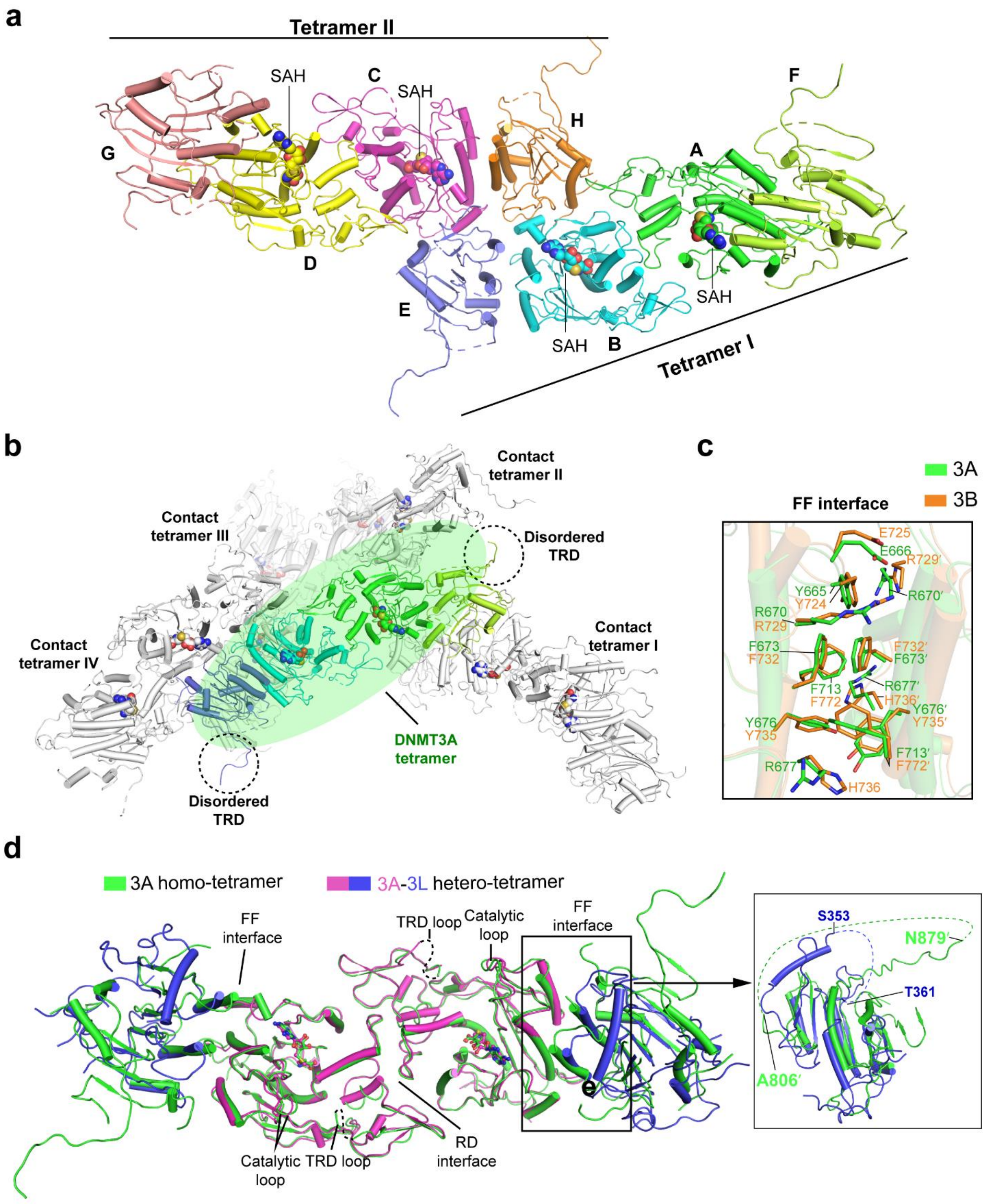

Supplementary Fig. 1. Structure determination of DNMT3A homotetramer. (A) DNMT3A molecules in one asymmetry unit of crystals. Note that the tetramer formed by monomer A, B, E and F was randomly selected for structural analysis. (B) Crystallization

contact analysis of DNMT3A homotetramer. The tetramer subject to analysis is shaded in green. The disordered TRD regions for the external subunits are highlighted in dashed circle. (C) Close-up view of the FF interfaces in DNMT3A homotetramer and DNMT3B homotetramer, with residues involved in intermolecular interactions show in stick representation. Hydrogen bonds are shown as dashed lines. (D) Structural overlay of DNMT3A homotetramer (green) and DNA-free mouse Dnmt3A (magenta)-Dnmt3L (slate) heterotetramer (PDB 2QRV), with the DNA-binding elements labeled. The disordered regions are indicated by dashed lines. The structural overlay between the external subunit of DNMT3A and DNMT3L C-terminal domain are shown in expanded view.

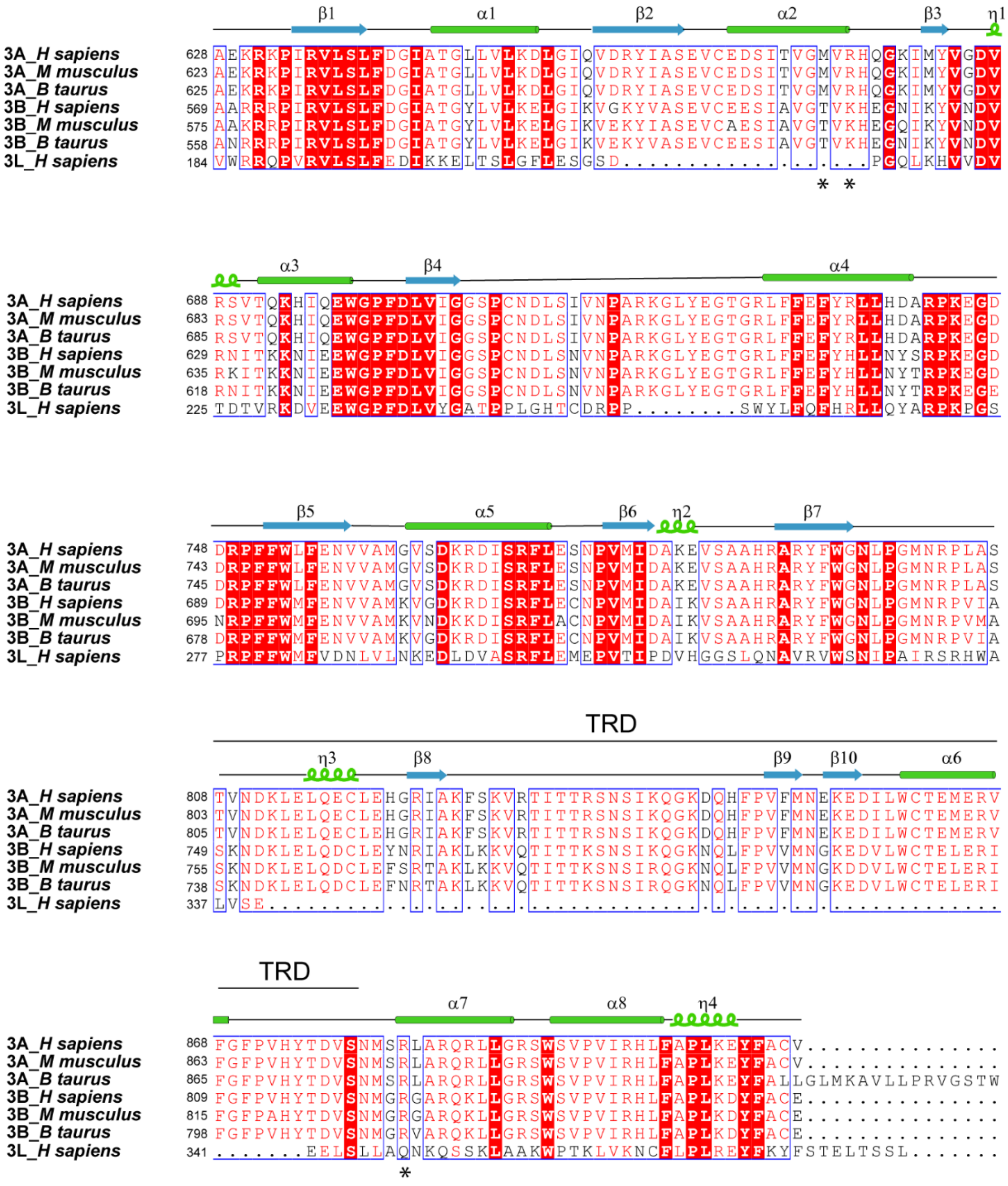

Supplementary Fig. 2. Structure-based sequence alignment of DNMT3A and DNMT3B MTase domains. Strictly conserved residues are colored white in the red background. Similar residues are colored red in the white background. The secondary structures of DNMT3A MTase domain are indicated above. The TRD embedded in the MTase domain is also indicated. DNMT3A M674- and R676-corresponding sites that are invariant in DNMT3A but replaced by threonine and lysine, respectively, in DNMT3B are marked by asterisks.

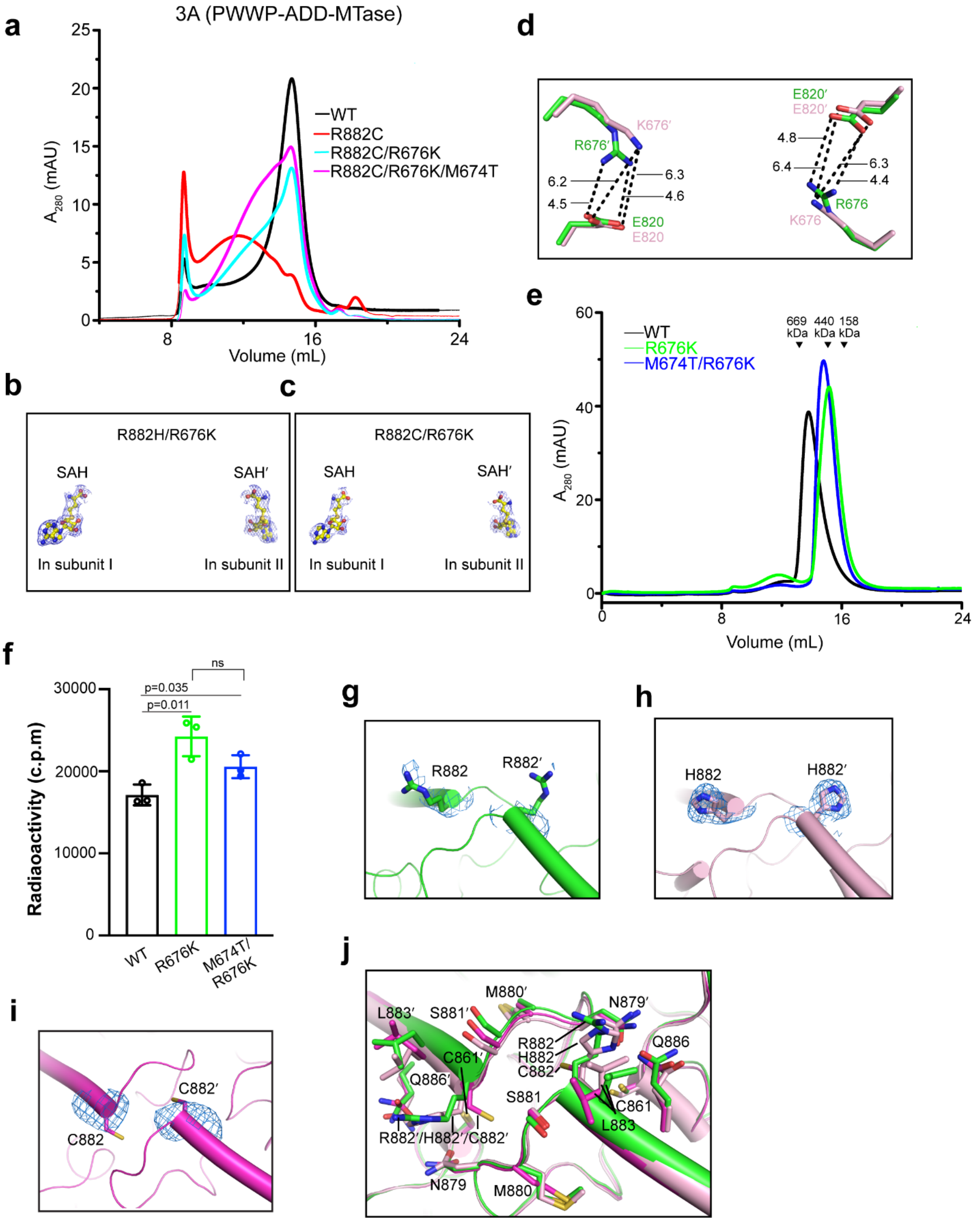

Supplementary Fig. 3. Structural and biochemical analysis of DNMT3A R882- and DNMT3B-converting mutations. (**a**) Size-exclusion chromatography analysis of DNMT3A fragment comprised of the PWWP, ADD and MTase domains, WT, R882C or

R882C/R676K and R882C/R676K/M674T. (**b**,**c**) Fo-Fc omit maps (blue; 2.0σ) the SAH molecules bound to DNMT3A$^{R882H/R676K}$ (b) and DNMT3A$^{R882C/R676K}$ (c). (**d**) Structural comparison of DNMT3A R676-E820′ ion pair and the corresponding pairs in DNMT3A$^{R882H/R676K}$. The side-chain distance for each pair is labeled. **(e)** Size-exclusion chromatography analysis of the MBP-tagged MTase domain of DNMT3A, WT (black), R676K (green) and M674T/R676K (blue). Elution volume for molecular weight standard was marked. **(f)** *In vitro* DNA methylation assays for DNMT3A MTase domain, either WT, R676K or M674T/R676K mutant. Statistical analysis used two-tailed Student's t-test. Data are mean$\pm$SD (n = 3 biological replicates). ns, not significant. Source data are provided as a Source Data file. (**g-i**) Fo-Fc omit map (blue; 2.0σ) of DNMT3A R882-corresponding site, shown in stick representation, in WT DNMT3A (green) (g), DNMT3A$^{R882H/R676K}$ (pink) (h) and DNMT3A$^{R882C/R676K}$ (magenta) (i). (**j**) Structural comparison between WT DNMT3A (green) (PDB 2QRV), DNMT3A$^{R882H/R676K}$ (pink) and DNMT3A$^{R882C/R676K}$ (magenta).

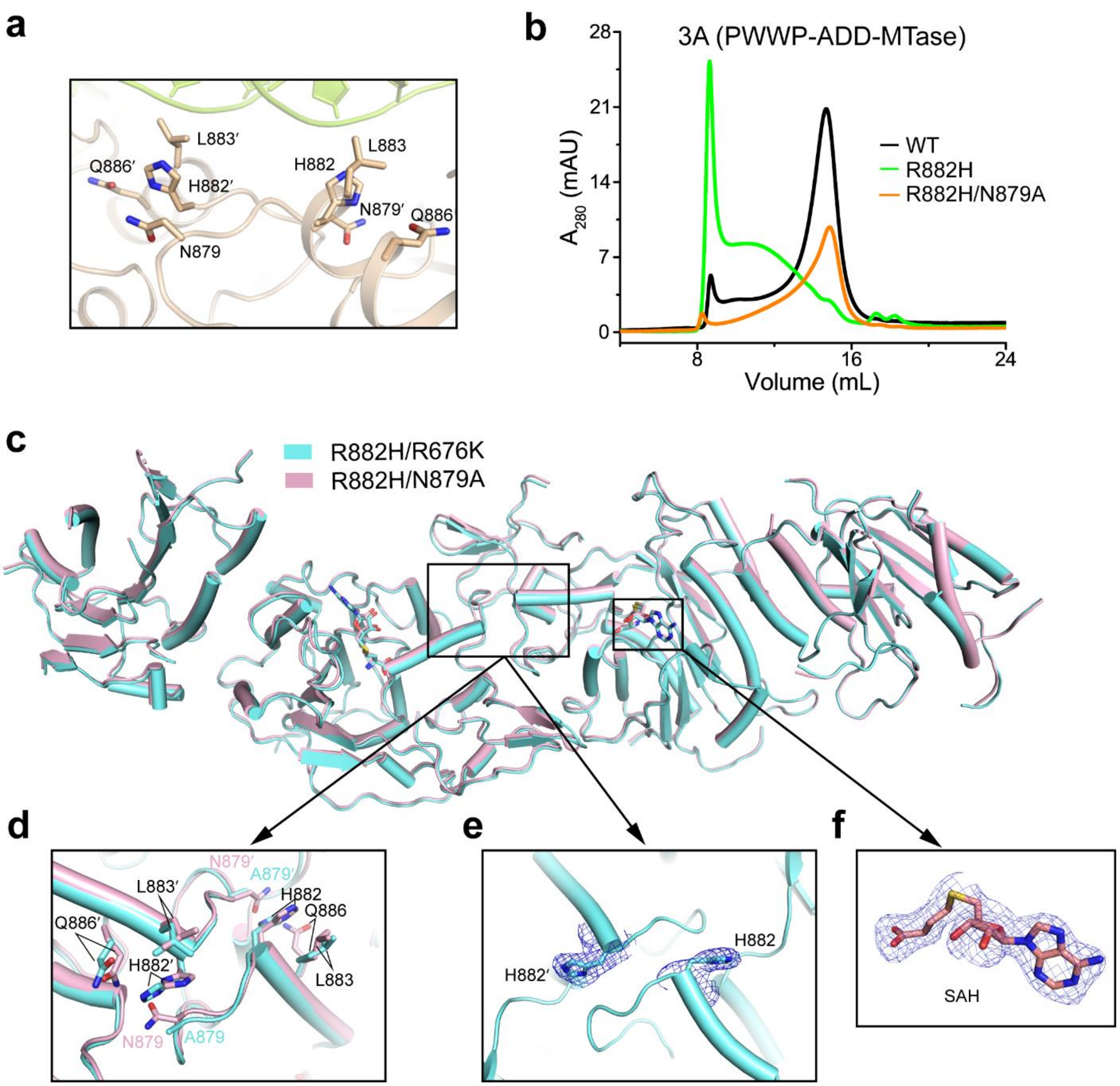

Supplementary Fig. 4. Strucutral and biochemical analysis of DNMT3A$^{R882H/N879A}$. (**a**) Close-up view of residues H882-mediated contacts in the DNMT3A^{R882H}-DNMT3L-DNA complex (PDB 6W8D). (**b**) Size-exclusion chromatography analysis of WT and R882H- and R882H/R676K -mutated DNMT3A fragments, comprised of the PWWP, ADD and MTase domains. (**c**) Structural overlay DNMT3A$^{R882H/N879A}$ and DNMT3A$^{R882H/R676K}$. (**d**) Close-up view of residue H882-mediated contacts in the DNMT3A$^{N879A/R882H}$-DNMT3L-DNA complex. (**e**) The Fo-Fc omit map (blue) of residue H882 in DNMT3A$^{R882H/N879A}$ is contoured at 2.0σ level. (**f**) The Fo-Fc omit map (slate) of the SAH molecule (salmon) bound to DNMT3A$^{R882H/N879A}$, contoured at 2.0σ level.

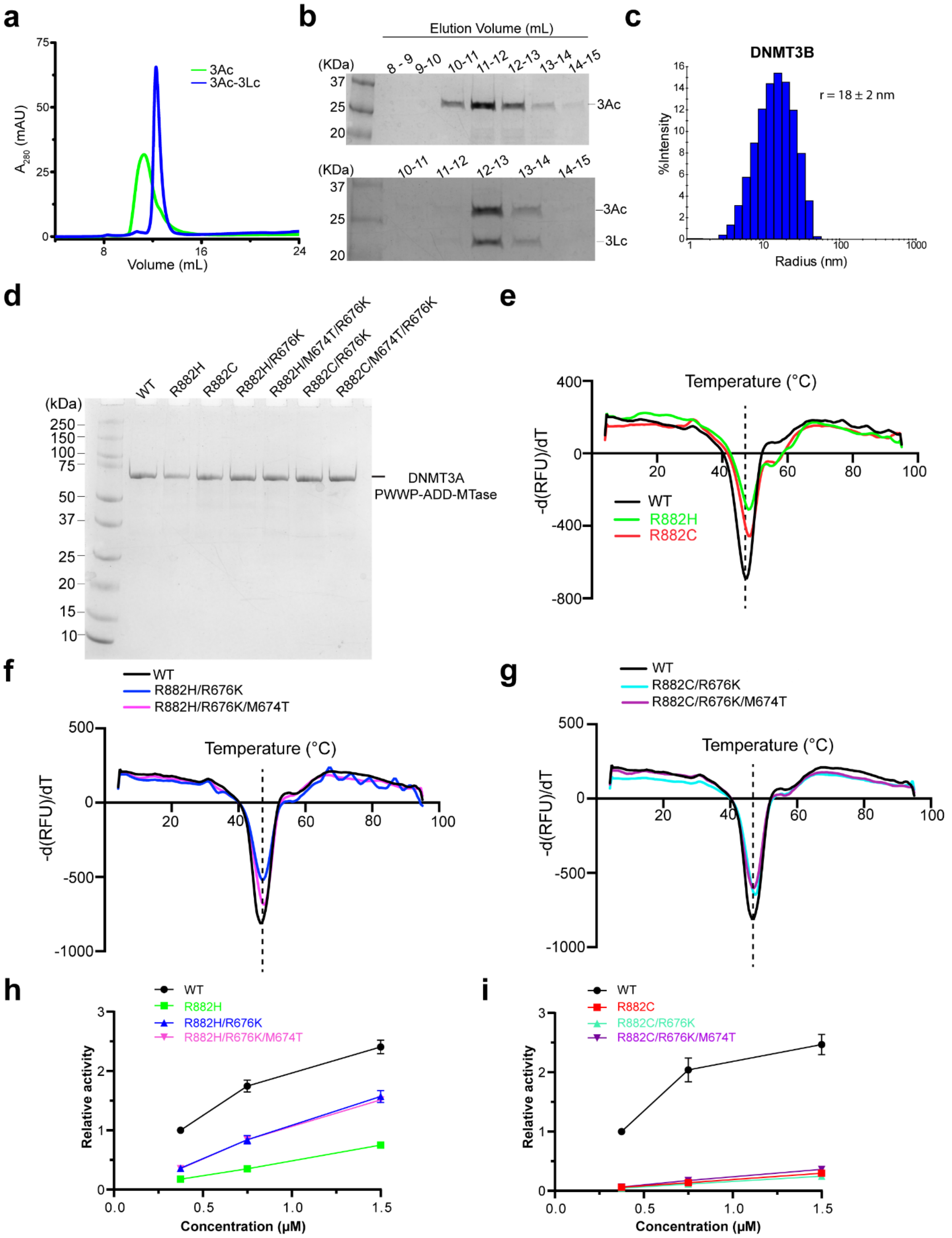

a
3Ac
3Ac-3Lc
A280 (mAU)
Volume (mL)
b
Elution Volume (mL)
(KDa)
8 - 9
9-10
10-11
11-12
12-13
13-14
14-15
3Ac
3Lc
c
DNMT3B
r = 18 ± 2 nm
%Intensity
Radius (nm)
d
WT
R882H
R882C
R882H/R676K
R882H/M674T/R676K
R882C/R676K
R882C/M674T/R676K
(kDa)
DNMT3A
PWWP-ADD-MTase
e
Temperature (°C)
-d(RFU)/dT
WT
R882H
R882C
f
WT
R882H/R676K
R882H/R676K/M674T
Temperature (°C)
-d(RFU)/dT
g
WT
R882C/R676K
R882C/R676K/M674T
Temperature (°C)
-d(RFU)/dT
h
WT
R882H
R882H/R676K
R882H/R676K/M674T
Relative activity
Concentration (μM)
i
WT
R882C
R882C/R676K
R882C/R676K/M674T
Relative activity
Concentration (μM)

Supplementary Fig. 5. Biochemical analysis of DNMT3A and DNMT3B MTase domains, WT or mutant. (**a**) Size-exclusion chromatography analysis of the C-terminal MTase domain of DNMT3A (3Ac) in homo-oligomeric form (green) or in complex with the C-terminal domain of DNMT3L (3Ac-3Lc) (blue). (**b**) SDS-PAGE images of corresponding fractions in (a), with elution volume marked on top. (**c**) DLS plot of MBP-tagged DNMT3B MTase domain. (**d**) SDS-PAGE images of DNMT3A PWWP-ADD-MTase fragments, wild-type (WT) and mutants, used for the thermal shift assay. (**e-g**) Comparison of the first-order derivatives of the raw fluorescence data for WT with that of R882H or R882C mutant (e), R882H/R676K or R882H/R676K/M674T mutant (f), and R882C/R676K or R882C/R676K/M674T mutant (g). (**h**) Relative DNA methylation activity of WT DNMT3A (n = 6 biological replicates), $DNMT3A^{R882H}$ (n = 6 biological replicates), $DNMT3A^{R882H/R676K}$ (n = 3 biological replicates) and $DNMT3A^{R882H/M674T/R676K}$ (n = 3 biological replicates) in a concentration-dependent manner. (**i**) Relative DNA methylation activity of WT DNMT3A (n = 6 biological replicates), $DNMT3A^{R882C}$ (n = 6 biological replicates), $DNMT3A^{R882C/R676K}$ (n = 3 biological replicates) and $DNMT3A^{R882C/M674T/R676K}$ (n = 3 biological replicates) in a concentration-dependent manner. Source data are provided as a Source Data file.

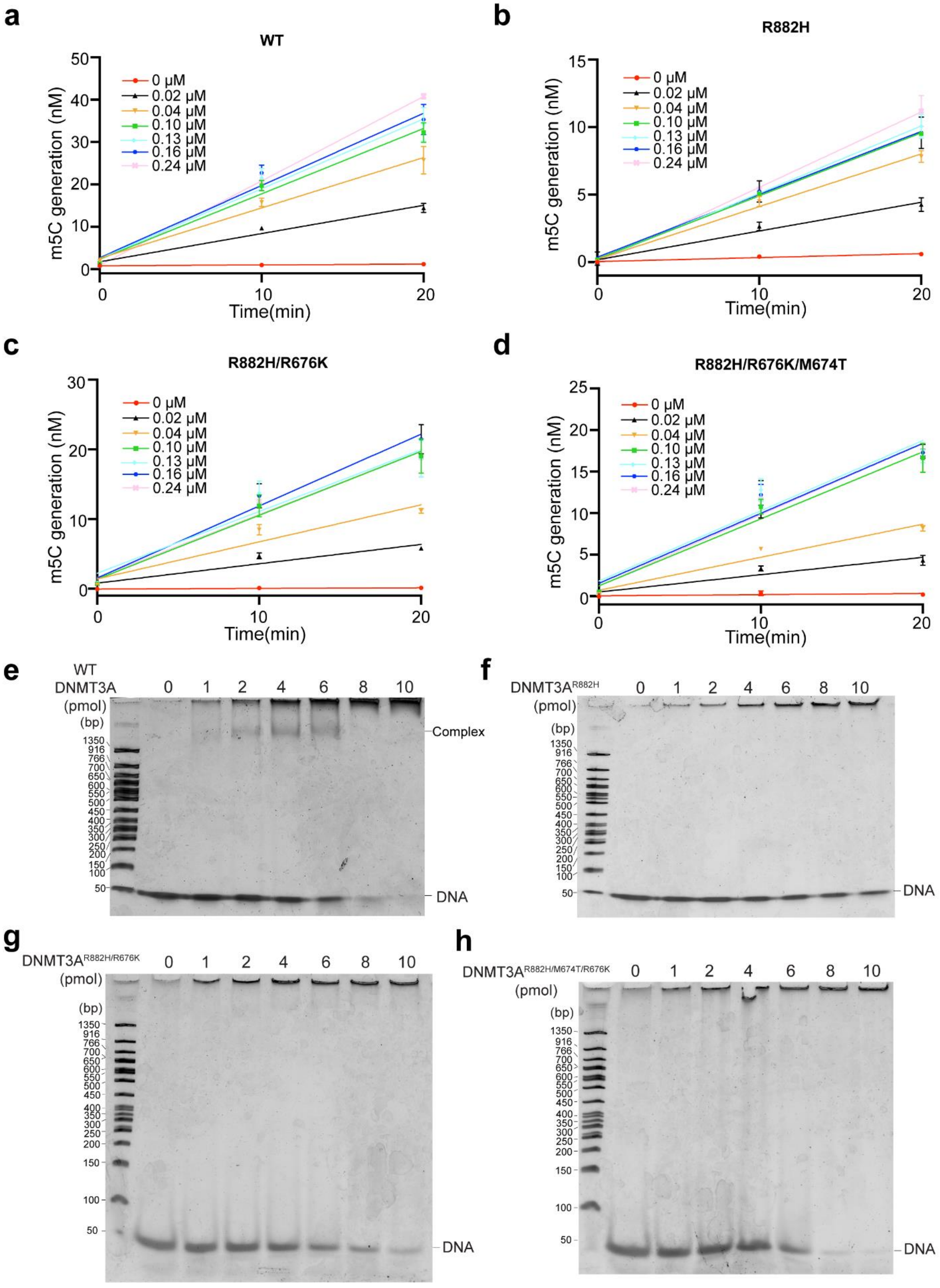
a
WT
m5C generation (nM)
Time(min)
0 μM
0.02 μM
0.04 μM
0.10 μM
0.13 μM
0.16 μM
0.24 μM
b
R882H
c
R882H/R676K
d
R882H/R676K/M674T
e
WT DNMT3A
(pmol)
(bp)
0 1 2 4 6 8 10
Complex
DNA
f
DNMT3A^R882H
g
DNMT3A^R882H/R676K
h
DNMT3A^R882H/M674T/R676K

Supplementary Fig. 6. *In vitro* DNA-methylation and binding assays for WT or mutant DNMT3A. (a-d) DNA methylation kinetics for MBP-tagged DNMT3A MTase domain, WT (a), R882H (b), R882H/R676K (c), or R882H/R676K/M674T mutant (d). Data are mean ± SD. (n = 3 biological replicates). (e-h) The EMSA result of the DNMT3A-DNA binding, WT or mutant. Increased concentration of 24-bp $(GAC)_8$ DNA duplex were incubated with MBP-tagged MTase domain of WT (e), R882H (f), R882H/R676K (g), and R882H/R676K/M674T (h). The experiment was performed twice with consistent results. Source data are provided as a Source Data file.

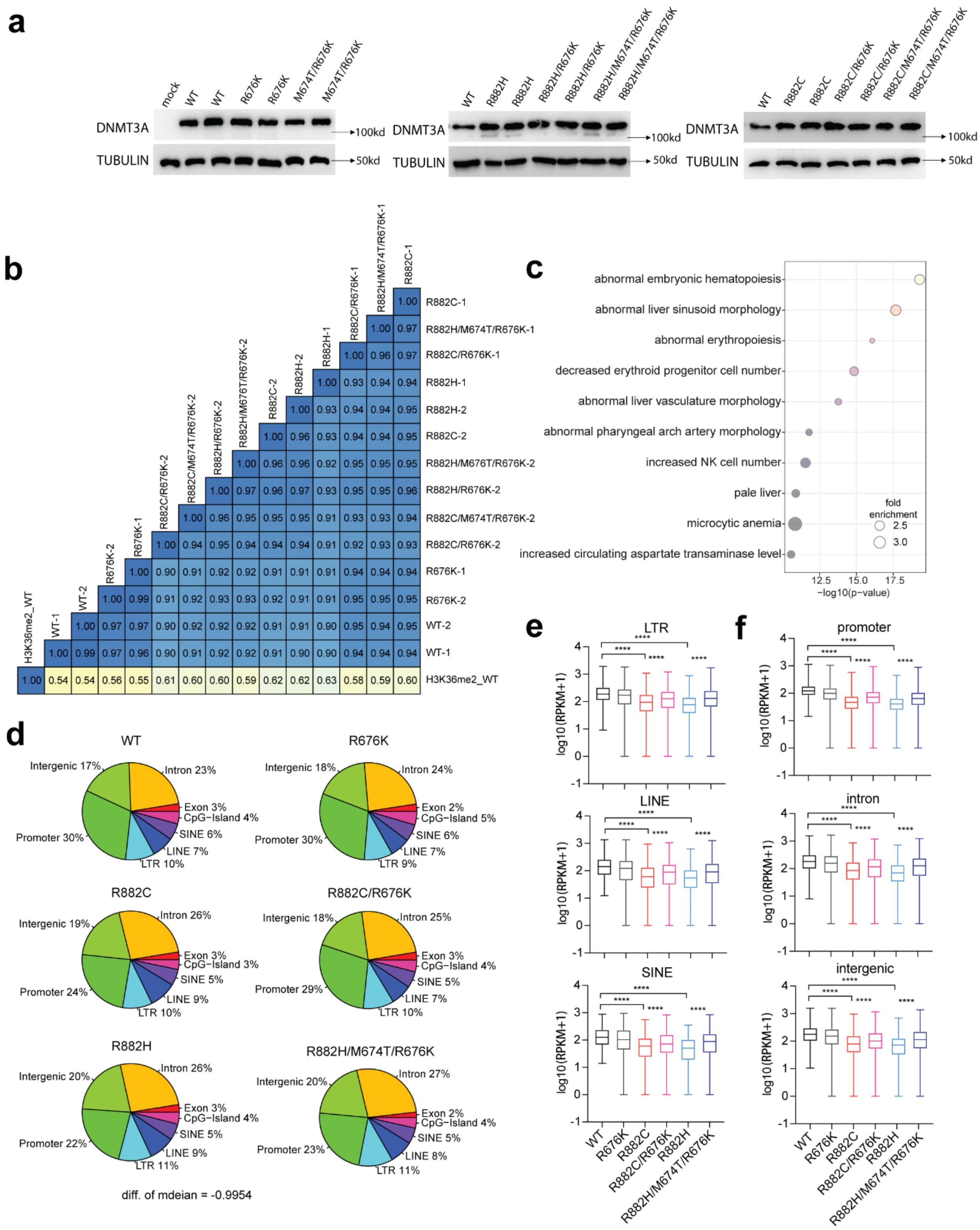
a
mock
WT
R676K
M674T/R676K
DNMT3A
TUBULIN
100kd
50kd
R882H
R882H/R676K
R882H/M674T/R676K
R882C
R882C/R676K
R882C/M674T/R676K
b
H3K36me2_WT
WT-1
WT-2
R676K-2
R676K-1
R882C/R676K-2
R882C/M674T/R676K-2
R882H/R676K-2
R882H/M676T/R676K-2
R882C-2
R882H-2
R882H-1
R882C/R676K-1
R882H/M674T/R676K-1
R882C-1
1.00 R882C-1
1.00 0.97 R882H/M674T/R676K-1
1.00 0.96 0.97 R882C/R676K-1
1.00 0.93 0.94 0.94 R882H-1
1.00 0.93 0.94 0.94 0.95 R882H-2
1.00 0.96 0.93 0.94 0.94 0.95 R882C-2
1.00 0.96 0.96 0.92 0.95 0.95 0.95 R882H/M676T/R676K-2
1.00 0.97 0.96 0.97 0.93 0.95 0.95 0.96 R882H/R676K-2
1.00 0.96 0.95 0.95 0.95 0.91 0.93 0.93 0.94 R882C/M674T/R676K-2
1.00 0.94 0.95 0.94 0.94 0.94 0.91 0.92 0.93 0.93 R882C/R676K-2
1.00 0.90 0.91 0.92 0.92 0.91 0.91 0.91 0.94 0.94 0.94 R676K-1
1.00 0.99 0.91 0.92 0.93 0.93 0.92 0.91 0.91 0.95 0.95 0.95 R676K-2
1.00 0.97 0.97 0.90 0.92 0.92 0.92 0.91 0.91 0.90 0.95 0.94 0.95 WT-2
1.00 0.99 0.97 0.96 0.90 0.91 0.92 0.92 0.91 0.90 0.90 0.94 0.94 0.94 WT-1
1.00 0.54 0.54 0.56 0.55 0.61 0.60 0.60 0.59 0.62 0.62 0.63 0.58 0.59 0.60 H3K36me2_WT
c
abnormal embryonic hematopoiesis
abnormal liver sinusoid morphology
abnormal erythropoiesis
decreased erythroid progenitor cell number
abnormal liver vasculature morphology
abnormal pharyngeal arch artery morphology
increased NK cell number
pale liver
microcytic anemia
increased circulating aspartate transaminase level
fold enrichment
2.5
3.0
12.5
15.0
17.5
−log10(p−value)
d
WT
Intergenic 17%
Intron 23%
Exon 3%
CpG−Island 4%
SINE 6%
LINE 7%
LTR 10%
Promoter 30%
R676K
Intergenic 18%
Intron 24%
Exon 2%
CpG−Island 5%
SINE 6%
LINE 7%
LTR 9%
Promoter 30%
R882C
Intergenic 19%
Intron 26%
Exon 3%
CpG−Island 3%
SINE 5%
LINE 9%
LTR 10%
Promoter 24%
R882C/R676K
Intergenic 18%
Intron 25%
Exon 3%
CpG−Island 4%
SINE 5%
LINE 7%
LTR 10%
Promoter 29%
R882H
Intergenic 20%
Intron 26%
Exon 3%
CpG−Island 4%
SINE 5%
LINE 9%
LTR 11%
Promoter 22%
R882H/M674T/R676K
Intergenic 20%
Intron 27%
Exon 2%
CpG−Island 4%
SINE 5%
LINE 8%
LTR 11%
Promoter 23%
diff. of mdeian = -0.9954
e
LTR
LINE
SINE
f
promoter
intron
intergenic
log10(RPKM+1)
****
WT
R676K
R882C
R882C/R676K
R882H
R882H/M674T/R676K

Supplementary Fig. 7. Rescue DNMT3A hotspot mutation-induced decrease of chromatin binding. (**a**) Western blot for the indicated Myc-tagged DNMT3A, WT or mutant, ectopically transduced in TF-1 cells. (**b**) Summary of Pearson's correlation coefficient between the indicated DNMT3A (WT or mutant; Myc-tagged) and H3K36me2 in TF-1 cells. DNMT3A CUT&Tag was performed with Myc antibody in the corresponding stable expression cell lines and H3K36me2 CUT&Tag performed in cells with WT DNMT3A. (**c**) GREAT analysis of the called WT DNMT3A CUT&Tag peaks reveals the enrichment of gene pathways associated with the indicated phenotype or cellular process. (**d**) Averaged intensity of binding by WT or mutant DNMT3A at the indicated repetitive element class, either LTR, LINEs or SINEs, in TF-1 cells as assayed by CUT&Tag and spike-in-control normalization. X-axis, ±5Kb from the start and end of the called peak. Y-axis, the median value of reads per kilobase per million reads mapped (RPKM). (**e**,**f**) Averaged intensity of binding by WT or mutant DNMT3A at various repetitive element classes (panel e; long terminal repeat (LTR), long interspersed nuclear elements (LINEs) or short interspersed nuclear elements (SINEs)), or at the genic (promoter, exon and intron) and intergenic regions (panel f), in TF-1 cells as assayed by CUT&Tag and spike-in-control normalization. The two-tailed Wilcoxon test was used for the comparison. ****$P < 0.0001$. Panels e,f and those shown in main Fig. 5b,c show results from the two independent experiments. Source data are provided as a Source Data file.

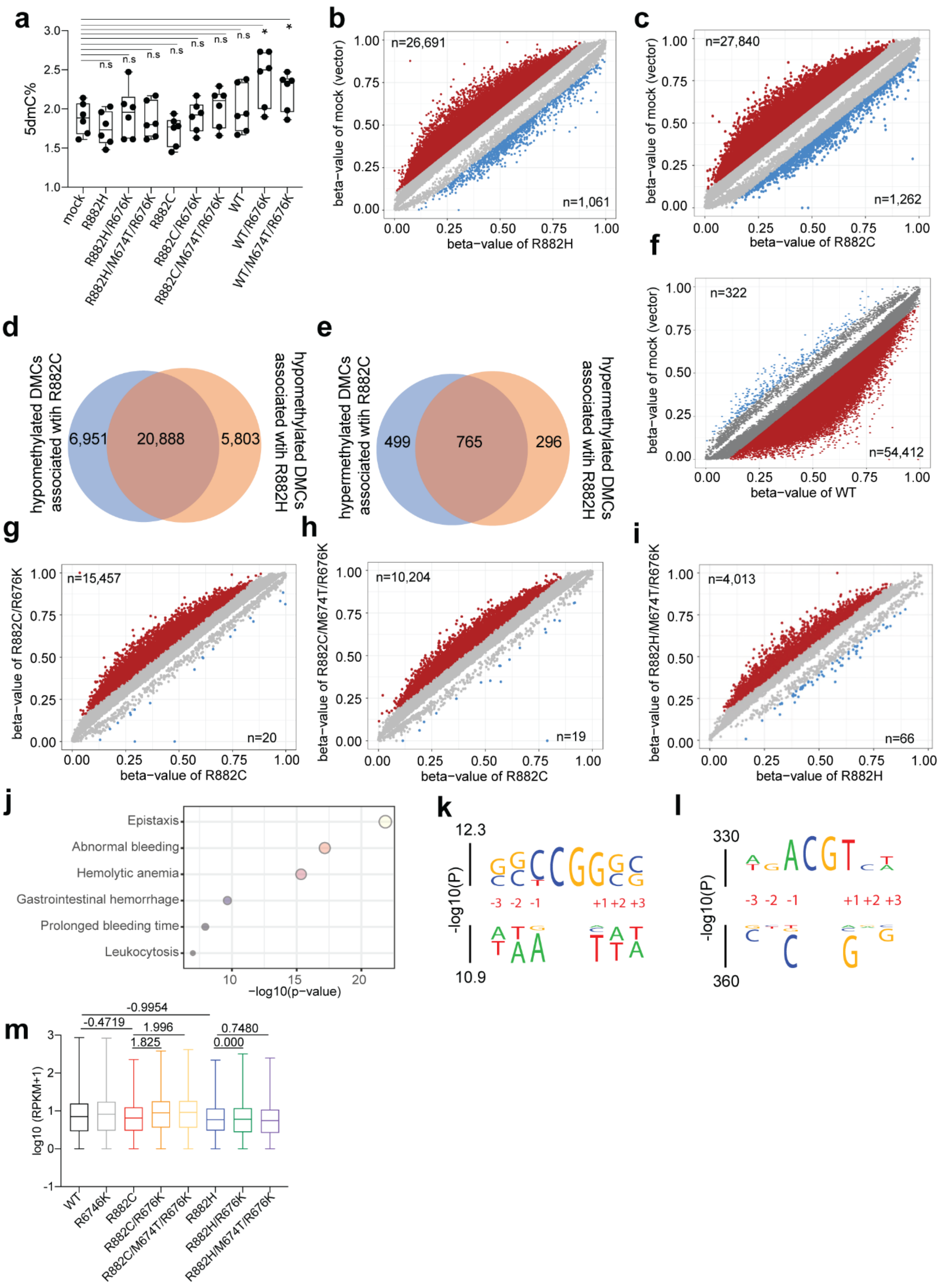
a
5dmC%
n.s
*
mock
R882H
R882H/R676K
R882H/M674T/R676K
R882C
R882C/R676K
R882C/M674T/R676K
WT
WT/R676K
WT/M674T/R676K
b
n=26,691
n=1,061
beta−value of mock (vector)
beta−value of R882H
c
n=27,840
n=1,262
beta−value of mock (vector)
beta−value of R882C
d
hypomethylated DMCs associated wtih R882C
6,951
20,888
5,803
hypomethylated DMCs associated wtih R882H
e
hypermethylated DMCs associated wtih R882C
499
765
296
hypermethylated DMCs associated wtih R882H
f
n=322
n=54,412
beta−value of mock (vector)
beta−value of WT
g
n=15,457
n=20
beta−value of R882C/R676K
beta−value of R882C
h
n=10,204
n=19
beta−value of R882C/M674T/R676K
beta−value of R882C
i
n=4,013
n=66
beta−value of R882H/M674T/R676K
beta−value of R882H
j
Epistaxis
Abnormal bleeding
Hemolytic anemia
Gastrointestinal hemorrhage
Prolonged bleeding time
Leukocytosis
−log10(p−value)
k
12.3
10.9
-log10(P)
-3 -2 -1
+1 +2 +3
l
330
360
-log10(P)
-3 -2 -1
+1 +2 +3
m
log10 (RPKM+1)
-0.9954
-0.4719
1.996
1.825
0.7480
0.000
WT
R6746K
R882C
R882C/R676K
R882C/M674T/R676K
R882H
R882H/R676K
R882H/M674T/R676K

Supplementary Fig. 8. Enhanced oligomerization of the DNMT3A hotspot mutant underlies the mutant-induced CpG hypo-methylation and cytokine-independent growth in TF-1 cells. (**a**) Mass spectrometry-based quantification of global methyl-cytosine levels in TF-1 cells ectopically expressed with the indicated DNMT3A, either WT or mutant, in comparison to mock-treated (vector). *, $p < 0.05$; ns, not significant. (**b**,**c**) Scatter plots showing the differentially methylated CpGs (DMCs) in TF-1 cells transduced with a hotspot mutation, R882H (panel b) or R882C (panel c), in comparison to mock. The cut-off of DMC is set at q value less than 0.01 (paired t-test) and △beta value greater than 0.1. Red and blue highlight DMCs exhibiting hypo-methylation and hyper-methylation, respectively, in cells with hotspot mutation relative to mock. (**d**,**e**) Venn diagram using the DMCs, either hypo-methylated (panel d) or hyper-methylated (panel e), in TF-1 cells transduced with DNMT3A R882C (left) or R882H (right) in comparison to mock (q value less than 0.01 in paired t-test and △beta value greater than 0.1). (**f**-**i**) Scatter plots showing DMCs in TF-1 cells transduced with WT DNMT3A in comparison to mock (panel f), DNMT3A R882C/R676K (panel g) or R882C/M674T/R676K (panel h) in comparison to R882C, or R882H/M674T/R676K in comparison to R882H (panel i). The cut-off of DMC is set at q value less than 0.01 (paired t-test) and △beta value greater than 0.1. (**j**) GREAT analysis using 20,888 DMCs defined in panel e uncovered significant enrichment in the signatures associated with the indicated phenotype or cellular process. (**k**,**l**) Flanking sequence preference using the hyper-methylated DMCs (k) (765 defined in panel e) and hypo-methylated (l) (20,888 defined in panel d) associated with DNMT3A hotspot mutation in comparison to mock in TF-1 cells. (**m**) Averaged intensity of binding by the indicated WT or mutant DNMT3A at DMCs exhibiting hypomethylation upon transduction of the R882 hotspot mutation in TF-1 cells (20,888; defined in panel d), as assayed by CUT&Tag and spike-in-control normalization. X-axis, ±5Kb from the DMC. Y-axis, the median value of reads per kilobase per million reads mapped (RPKM; ± 50bp from the CpG site). The numbers shown on top of the columns refer to difference in the median value of RPKM between the sample with R882-mutated DNMT3A versus WT, or the sample with a converting mutant versus that without.

Supplementary Table 1. Crystallographic data collection and refinement statistics

Code	WT<br>PDB: 8TDR	R882H/R676K<br>PDB: 8TE1	R882C/R676K<br>PDB: 8TE3	R882H/N879A<br>PDB: 8TE4
**Data collection**				
Space group	*P 3*	*P 3*	*P 3*	*P 3*
Cell dimensions				
a, *b*, *c* (Å)	179.4,179.4,108	177.8,177.8,110.9	177.3,177.3,110.7	178.1, 178.1, 109.9
α, β, γ (°)	90,90,120	90,90,120	90,90,120	90, 90, 120
Wavelength	0.97648	0.97648	0.97648	0.97648
Resolution (Å)	46.29-3.32<br>(3.44-3.32) [a]	47.06-2.48<br>(2.57-2.48) [a]	46.46-3.2<br>(3.31-3.2) [a]	46.56-2.65<br>(2.74-2.65) [a]
R_{merge}	0.219(1.326)	0.155(1.94)	0.29(1.281)	0.155 (1.551)
$I / \sigma I$	5.5(0.8)	7.8(0.6)	4.8(0.8)	10.2 (1.1)
$CC_{1/2}$	0.985(0.834)	0.995(0.3)	0.96(0.324)	0.995 (0.339)
Completeness (%)	99.4(98.4)	98.9(96.6)	98.8(98.1)	99.4 (98.6)
Redundancy	3.9(3.7)	5.8(5.6)	4.3(4.2)	5.5 (5.3)
Total reflections	225050	803976	273523	619789
Unique reflections	57295	138899	64243	113210
**Refinement**				
No. reflections	57058	137486	63504	112538
R_{work} / R_{free} (%)	21.0/23.8	19.2/22.4	20.7/24.1	20.2/23.5
No. atoms				
Protein	14327	14039	13981	14061
SAH	104	104	104	104
B factors (Å^2)				
Protein	81.08	60.03	56.47	58.78
SAH	75.66	49.16	45.8	50.92
r.m.s. deviations				
Bond lengths (Å)	0.003	0.003	0.003	0.003
Bond angles (°)	0.64	0.65	0.60	0.64
Ramachandran				
Favored (%)	96.66	97.75	96.88	96.77
Allowed (%)	3.34	2.25	3.12	3.11
Outliers (%)	0	0	0	0.12

[a]Values in parentheses are for highest-resolution shell. Each dataset was collected from a single crystal.
