# Supplementary material for: Structure-guided functional suppression of AML-associated DNMT3A hotspot mutations
Source: Nat Commun. 2024 Apr 10;15:3111. doi: 10.1038/s41467-024-47398-y (PMC11006857; doi:10.1038/s41467-024-47398-y)
Supplement: Supplementary file 3 — Reporting Summary [file 41467_2024_47398_MOESM3_ESM.pdf]

nature portfolio

Corresponding author(s): Jikui Song ,Gang Greg Wang

Last updated by author(s): Mar 6, 2024

Reporting Summary

Nature Portfolio wishes to improve the reproducibility of the work that we publish. This form provides structure for consistency and transparency in reporting. For further information on Nature Portfolio policies, see our Editorial Policies and the Editorial Policy Checklist.

Statistics

For all statistical analyses, confirm that the following items are present in the figure legend, table legend, main text, or Methods section.

n/a	Confirmed	
☐	☒	The exact sample size (*n*) for each experimental group/condition, given as a discrete number and unit of measurement
☐	☒	A statement on whether measurements were taken from distinct samples or whether the same sample was measured repeatedly
☐	☒	The statistical test(s) used AND whether they are one- or two-sided<br>*Only common tests should be described solely by name; describe more complex techniques in the Methods section.*
☒	☐	A description of all covariates tested
☒	☐	A description of any assumptions or corrections, such as tests of normality and adjustment for multiple comparisons
☐	☒	A full description of the statistical parameters including central tendency (e.g. means) or other basic estimates (e.g. regression coefficient) AND variation (e.g. standard deviation) or associated estimates of uncertainty (e.g. confidence intervals)
☐	☒	For null hypothesis testing, the test statistic (e.g. *F*, *t*, *r*) with confidence intervals, effect sizes, degrees of freedom and *P* value noted<br>*Give P values as exact values whenever suitable.*
☒	☐	For Bayesian analysis, information on the choice of priors and Markov chain Monte Carlo settings
☒	☐	For hierarchical and complex designs, identification of the appropriate level for tests and full reporting of outcomes
☒	☐	Estimates of effect sizes (e.g. Cohen's *d*, Pearson's *r*), indicating how they were calculated

Our web collection on statistics for biologists contains articles on many of the points above.

Software and code

Policy information about availability of computer code

Data collection	X-ray diffraction data were collected using the standard data collection software from synchrotron beam lines 5.0.2 and 5.0. 3 at the Advanced Light Source, Lawrence Berkeley National Laboratory. Dynamic light scattering data were collected using DynaPro Plate Reader II (Wyatt Technology Corporation). Fluorescence data for thermo-shift assay was collected on CFX96 Connect Real-Time PCR system (Bio-rad).For SDS-PAGE and EMSA gel were scanned using ChemiDoc Imaging System (Bio-rad). WGBS data were collected on the NovaSeq 54 sequencer platform. Infinium MethylationEPIC microarray was sequenced using iScan system (illumina). CUT&Tag was sequenced by NEXTSEQ550 (illumina)
Data analysis	HKL3000 (v721.3),Phaser (v2.7.16), Coot:WinCoot 0.8.9 EL, Phenix (vl.20.1_ 4487), Pymol (v2.5.2), Origin (v7.0), GraphPad Prism (v10.0.1). Minfi(1.49.1) for Infinium methylationEPIC array. Samtools(1.9), Picard(2.22.4), Bedtools(2.12), DeepTools(v3.3.0), MACS(v2.1.1), Bowtie (2.3.4) for CUT&Tag analysis.The EzColocalization software is used for analysis of colocalization in cells.

For manuscripts utilizing custom algorithms or software that are central to the research but not yet described in published literature, software must be made available to editors and reviewers. We strongly encourage code deposition in a community repository (e.g. GitHub). See the Nature Portfolio guidelines for submitting code & software for further information.

Data

Policy information about availability of data

All manuscripts must include a data availability statement. This statement should provide the following information, where applicable:

- Accession codes, unique identifiers, or web links for publicly available datasets
- A description of any restrictions on data availability
- For clinical datasets or third party data, please ensure that the statement adheres to our policy

Coordinates and structural factors for methyltransferase domain of DNMT3A WT, R882H/R676K, R882C/R676K and R882H/N879A homotetramer are deposited in the PDB database under accession codes 8TDR, 8TE1,8TE3 and 8TE4).Human DNMT3A-DNMT3L-DNA complex (PDB 5YX2) was used as a search model for structure determination. Human DNMT3A/3L heterotetramer (PDB 2QRV),DNMT3A(R882H)-DNMT3L-DNA complex (PDB 6W8D) and DNMT3B homotetramer (PDB 8EIH) were used for structural comparison.
The Cut&Tag and DNA methylation profiling data have been deposited in NCBI Gene Expression Omnibus under accession number GSE225828 and GSE226062 respectively.

Research involving human participants, their data, or biological material

Policy information about studies with human participants or human data. See also policy information about sex, gender (identity/presentation), and sexual orientation and race, ethnicity and racism.

Reporting on sex and gender	N/A
Reporting on race, ethnicity, or other socially relevant groupings	N/A
Population characteristics	N/A
Recruitment	N/A
Ethics oversight	N/A

Note that full information on the approval of the study protocol must also be provided in the manuscript.

Field-specific reporting

Please select the one below that is the best fit for your research. If you are not sure, read the appropriate sections before making your selection.

[x] Life sciences [ ] Behavioural & social sciences [ ] Ecological, evolutionary & environmental sciences

For a reference copy of the document with all sections, see nature.com/documents/nr-reporting-summary-flat.pdf

Life sciences study design

All studies must disclose on these points even when the disclosure is negative.

Sample size	Sample size was chosen to ensure reproducibility of the results at affordable costs. At least 3 replicates were carried for in vitro enzymatic assays. The dynamic light scattering measurement was repeated three times with consistent results. Thermo-shift assay were performed with triplicate samples. Electrophoresis mobility shift assay was repeated twice with consistent result. 3-4 replicates of each DNMT3A mutations were used for Infinium methylationEPIC array. 2 replicates were used for CUT&Tag assay.
Data exclusions	No data were excluded from analysis.
Replication	All attempts at replication were successful and noted in the relevant figure legends.
Randomization	No randomization techniques were required since we did not use different experimental groups or conditions in our study.
Blinding	Blinding was not performed due to the unambiguous nature of measurements and systematic analyses used in these experiments.

Reporting for specific materials, systems and methods

We require information from authors about some types of materials, experimental systems and methods used in many studies. Here, indicate whether each material, system or method listed is relevant to your study. If you are not sure if a list item applies to your research, read the appropriate section before selecting a response.

Materials & experimental systems

n/a	Involved in the study	
☐	☒	Antibodies
☐	☐	Eukaryotic cell lines
☒	☐	Palaeontology and archaeology
☒	☐	Animals and other organisms
☒	☐	Clinical data
☒	☐	Dual use research of concern
☒	☐	Plants

Methods

n/a	Involved in the study	
☐	☒	ChIP-seq
☒	☐	Flow cytometry
☒	☐	MRI-based neuroimaging

Antibodies

Antibodies used	Antibodies used for<br>1. immunoblotting (1:1000 dilution)<br>Anti-Dnmt3a antibody (abcam ab2850)<br>β-Tubulin Antibody (Cell Signalling #2146)<br>Anti-rabbit IgG, HRP-linked Antibody (Cell Signalling #7074)<br>2. CUT&Tag (1:50 dilution)<br>Myc-Tag (9B11) Mouse mAb (Cell Signalling #2276)<br>Anti-Histone H3 (tri methyl K9) antibody - ChIP Grade (Abcam ab8898)<br>Histone H3K36me2 antibody (Active Motif 39255)<br>Anti-Mouse Secondary Antibody for CUTANA™ CUT&Tag Workflows (EpiCypher 13-0048)<br>Anti-Rabbit Secondary Antibody for CUTANA™ CUT&Tag Workflows (EpiCypher 13-0047)
Validation	All antibodies used are commercially available and validated by corresponding companies.<br>Anti-Dnmt3a antibody (abcam ab2850) (https://www.abcam.com/products/primary-antibodies/dnmt3a-antibody-ab2850.html)<br>β-Tubulin Antibody (Cell Signalling #2146) (https://www.cellsignal.com/products/primary-antibodies/b-tubulin-antibody/2146)<br>Anti-rabbit IgG, HRP-linked Antibody (Cell Signalling #7074) (https://www.cellsignal.com/products/secondary-antibodies/anti-rabbit-igg-hrp-linked-antibody/7074)<br>Myc-Tag (9B11) Mouse mAb (Cell Signalling #2276) (https://www.cellsignal.com/products/primary-antibodies/myc-tag-9b11-mouse-mab/2276)<br>Anti-Histone H3 (tri methyl K9) antibody - ChIP Grade (Abcam ab8898) (https://www.abcam.com/products/primary-antibodies/histone-h3-tri-methyl-k9-antibody-chip-grade-ab8898.html)<br>Histone H3K36me2 antibody (Active Motif 39255) (https://www.activemotif.com/catalog/details/39255/histone-h3-dimethyl-lys36-antibody-pab)<br>Anti-Mouse Secondary Antibody for CUTANA™ CUT&Tag Workflows (EpiCypher 13-0048) (https://www.epicypher.com/products/epigenetics-reagents-and-assays/cutanac-cut-tag-assays/anti-mouse-secondary-antibody-for-cutana-cut-tag-workflows)<br>Anti-Rabbit Secondary Antibody for CUTANA™ CUT&Tag Workflows (EpiCypher 13-0047) (https://www.epicypher.com/products/antibodies/cutana-cut-tag-antibodies/anti-rabbit-secondary-antibody-for-cutana-cut-tag-workflows)

Eukaryotic cell lines

Policy information about cell lines and Sex and Gender in Research

Cell line source(s)	Human TF-1 erythroblasts cell line (ATCC #CRL-2003) was cultivated and utilized in this study.
Authentication	Authentication of cell line identity, including that of parental and derived lines, was ensured by Tissue Culture Facility affiliated to the Lineberger Comprehensive Cancer Center of UNC at Chapel Hill using the genetic signature profiling and fingerprinting analysis.
Mycoplasma contamination	Every 1-2 month, a routine examination of cell lines in culture for any possible mycoplasma contamination was carried out using MycoAlert Mycoplasma Detection Kit (Lonza). No mycoplasma contamination was identified.
Commonly misidentified lines<br>(See ICLAC register)	No commonly misidentified cell lines were used in the study.

Plants

Seed stocks

N/A

Novel plant genotypes

N/A

Authentication

N/A

ChIP-seq

Data deposition

- [x] Confirm that both raw and final processed data have been deposited in a public database such as GEO.
- [x] Confirm that you have deposited or provided access to graph files (e.g. BED files) for the called peaks.

Data access links
May remain private before publication.

https://www.ncbi.nlm.nih.gov/geo/query/acc.cgi?acc=GSE225828
https://www.ncbi.nlm.nih.gov/geo/query/acc.cgi?acc=GSE226062

Files in database submission

1/raw fastq files
TF1-WT_batch1-R1.fastq.gz
TF1-WT-K_batch1-R1.fastq.gz
TF1-R882H_batch1-R1.fastq.gz
TF1-HTK_batch1-R1.fastq.gz
TF1-CK_batch1-R1.fastq.gz
TF1-CTK_batch1-R1.fastq.gz
TF1-WT_batch1-R2.fastq.gz
TF1-WT-K_batch1-R2.fastq.gz
TF1-R882H_batch1-R2.fastq.gz
TF1-HTK_batch1-R2.fastq.gz
TF1-CK_batch1-R2.fastq.gz
TF1-CTK_batch1-R2.fastq.gz
TF1-WT_batch2-R1.fastq.gz
TF1-WT-K_batch2-R1.fastq.gz
TF1-R882H_batch2-R1.fastq.gz
TF1-HK_batch2-R1.fastq.gz
TF1-HTK_batch2-R1.fastq.gz
TF1-R882C_batch2-R1.fastq.gz
TF1-CK_batch2-R1.fastq.gz
TF1-CTK_batch2-R1.fastq.gz
TF1_H3K36me2_WT-R1.fastq.gz
TF1-WT_batch2-R1.fastq.gz TF1-WT_batch2-R2.fastq.gz
TF1-WT-K_batch2-R1.fastq.gz TF1-WT-K_batch2-R2.fastq.gz
TF1-R882H_batch2-R1.fastq.gz TF1-R882H_batch2-R2.fastq.gz
TF1-HK_batch2-R1.fastq.gz TF1-HK_batch2-R2.fastq.gz
TF1-HTK_batch2-R1.fastq.gz TF1-HTK_batch2-R2.fastq.gz
TF1-R882C_batch2-R1.fastq.gz TF1-R882C_batch2-R2.fastq.gz
TF1-CK_batch2-R1.fastq.gz TF1-CK_batch2-R2.fastq.gz
TF1-CTK_batch2-R1.fastq.gz TF1-CTK_batch2-R2.fastq.gz
TF1_H3K36me2_WT-R1.fastq.gz TF1_H3K36me2_WT-R2.fastq.gz
2/ processed bigwig files
TF1-WT_batch1.bw
TF1-WT-K_batch1.bw
TF1-R882H_batch1.bw
TF1-HTK_batch1.bw
TF1-R882C_batch1.bw
TF1-CK_batch1.bw
TF1-WT_batch2.bw
TF1-WT-K_batch2.bw
TF1-R882H_batch2.bw
TF1-HK_batch2.bw

	TF1-HTK_batch2.bw<br>TF1-R882C_batch2.bw<br>TF1-CK_batch2.bw<br>TF1-CTK_batch2.bw<br>TF1_H3K36me2_WT.bw
Genome browser session (e.g. UCSC)	no longer applicable

Methodology

Replicates	TF-1 cell line was stably expressed with Myc-tagged DNMT3A (isoform 1), either wild-type (WT) or AML-associated hotspot mutant (R882C or R882H) or the one carrying an additional macro-oligomerization-decreasing mutation, namely, R676K (WT-K), R882C/R676K (R882C/K), R882C/M674T/R676K (CTK), R882H/R676K (R882H/K) or R882H/M674T/R676K (R882H/TK). 100,000 of TF-1 cells were subjected to CUT&Tag using anti-Myc tag antibody (CST #2276s). 5,000 of NIH-3T3 cells with stable expression of Myc-tagged WT DNMT3A1 were added to all TF-1 cell groups as spike-in normalization control.
Sequencing depth	> 8 million of raw reads of each CUT&Tag sample
Antibodies	α-myc tag (Cell Signalling #2276) and H3K36me2 (Active Motif 39255)
Peak calling parameters	MACS2 with -f BAMPE --keep-dup 1 --cutoff-analysis
Data quality	FastQC
Software	DeepTools(v3.3.0), MACS(v2.1.1), Bowtie (2.3.4)
